# Development of Lactoferrin-Loaded Liposomes for the Management of Dry Eye Disease and Ocular Inflammation

**DOI:** 10.3390/pharmaceutics13101698

**Published:** 2021-10-15

**Authors:** Ana López-Machado, Natalia Díaz-Garrido, Amanda Cano, Marta Espina, Josefa Badia, Laura Baldomà, Ana Cristina Calpena, Eliana B. Souto, María Luisa García, Elena Sánchez-López

**Affiliations:** 1 Department of Pharmacy and Pharmaceutical Technology and Physical Chemistry, Faculty of Pharmacy and Food Sciences, University of Barcelona, 08028 Barcelona, Spain; alopezmachado@ub.edu (A.L.-M.); acanofernandez@ub.edu (A.C.); m.espina@ub.edu (M.E.); anacalpena@ub.edu (A.C.C.); 2 Institute of Nanoscience and Nanotechnology (IN2UB), University of Barcelona, 08028 Barcelona, Spain; 3 Department of Biochemistry & Physiology, Faculty of Pharmacy & Food Sciences, University of Barcelona, 08028 Barcelona, Spain; ndiazgarrido@ub.edu (N.D.-G.); josefabadia@ub.edu (J.B.); lbaldoma@ub.edu (L.B.); 4 Institute of Biomedicine (IBUB), University of Barcelona, 08028 Barcelona, Spain; 5 Sant Joan de Déu Research Institute (IR-SJD), 08950 Barcelona, Spain; 6 Biomedical Research Networking Centre in Neurodegenerative Diseases (CIBERNED), 28031 Madrid, Spain; 7 Centre of Biological Engineering (CEB), Campus de Gualtar, University of Minho, 4710-057 Braga, Portugal; eliana.souto@ceb.uminho.pt

**Keywords:** lactoferrin, liposomes, dry eye disease, ocular anti-inflammatory, drug delivery

## Abstract

Dry eye disease (DED) is a high prevalent multifactorial disease characterized by a lack of homeostasis of the tear film which causes ocular surface inflammation, soreness, and visual disturbance. Conventional ophthalmic treatments present limitations such as low bioavailability and side effects. Lactoferrin (LF) constitutes a promising therapeutic tool, but its poor aqueous stability and high nasolacrimal duct drainage hinder its potential efficacy. In this study, we incorporate lactoferrin into hyaluronic acid coated liposomes by the lipid film method, followed by high pressure homogenization. Pharmacokinetic and pharmacodynamic profiles were evaluated in vitro and ex vivo. Cytotoxicity and ocular tolerance were assayed both in vitro and in vivo using New Zealand rabbits, as well as dry eye and anti-inflammatory treatments. LF loaded liposomes showed an average size of 90 nm, monomodal population, positive surface charge and a high molecular weight protein encapsulation of 53%. Biopharmaceutical behaviour was enhanced by the nanocarrier, and any cytotoxic effect was studied in human corneal epithelial cells. Developed liposomes revealed the ability to reverse dry eye symptoms and possess anti-inflammatory efficacy, without inducing ocular irritation. Hence, lactoferrin loaded liposomes could offer an innovative nanotechnological tool as suitable approach in the treatment of DED.

## 1. Introduction

Dry eye disease (DED) or keratoconjunctivitis sicca is considered a chronic multifactorial pathology of the ocular surface characterized by a loss of homeostasis of the tear film, associated with characteristic ocular symptoms, such as tear film instability and hyperosmolarity, ocular surface inflammation and damage. Moreover, neurosensory abnormalities play etiological roles, according to the TFOS DEWS II [1].

DED is one of the most frequent ocular surface conditions, affecting millions of patients globally, with a prevalence ranging from 5 to 50% [2,3]. Numerous risk factors are identified, including advanced age, female gender, Sjögren syndrome, androgen deficiency, several medications such as antihistamines, antidepressants, anxiolytics, or oral contraceptives, thyroid disease, menopause and smoking, among others [4]. Moreover, the continuous use of contact lenses, certain environmental conditions as elevated pollution or low humidity, and the excessive smartphone and computer use has led to an increase in DED incidence especially among the younger population [4].

This osmotic and cellular stress at the ocular surface leads to irritation, ocular surface inflammation, soreness, blurred vision, and visual disturbance, resulting in a considerable decline in quality of life [3]. The elevated tear osmolarity, oxidative and mechanical stress-associated trigger a pro-inflammatory environment [5]. It is characterized by a broad release of pro-inflammatory mediators, cytokines, chemokines, and immune cells, leading to the extracellular matrix degradation and disruption of tight junctions between corneal epithelial cells. These conditions damage the ocular surface and favour inflammatory cell recruitment [6,7]. Thus, generating a self-inflammatory feedback loop that affects ocular function and integrity [4]. 

Topical administration is the preferred route to treat DED because it is painless and easy to handle. Artificial tears in the form of eyedrops, gel or ointment are used to lubricate dry eyes maintaining moisture of the eye’s surface and often constitute the first line of therapy. They instantly relieve symptoms by lowering osmolarity and diluting inflammatory markers. However, artificial tears have no anti-inflammatory properties and do not deal with the fundamental pathogenesis of the disease [4]. Moreover, usual treatments for ocular inflammation comprises corticosteroids and non-steroidal anti-inflammatory drugs (NSAIDs), but its prolonged use involves severe side effects [8,9,10].

To overcome these drawbacks, lactoferrin (LF), an iron-binding glycoprotein with anti-inflammatory, antiviral, antibacterial, antifungal, antiparasitic, and immunomodulatory properties, has been investigated to address various ocular disorders [11,12,13,14].

LF has two highly homologous lobes with stable and reversible iron-binding capacity. LF is secreted by neutrophils and exocrine glands and it is found in colostrum and milk, tears, saliva, or gastrointestinal secretions [15]. At ocular level, LF amount is around 20–30% in basal and reflex tears and is also present in vitreous humour and a variety of ocular tissues, such as cornea, iris, and retinal pigment epithelium [16,17]. Moreover, recent studies have confirmed that the concentration of LF in tear fluids is considerably lower in patients with DED [18].

Most of the in vitro and in vivo studies have been assayed using bovine LF (bLF) since presents high sequence homology and has analogous functions to human LF [19]. bLF is generally recognized as safe substance (GRAS) by the Food and Drug Administration (FDA) and the European Food Safety Authority (EFSA) [19,20]. bLF is internalized by corneal epithelial cells and exerts its anti-inflammatory activity by attenuating the nuclear transcription factor kappa B (NF-κB)-induced transcription of genes for several inflammatory mediators [19,21,22,23]. 

Furthermore, reactive oxygen species (ROS) also play a major role in inflammatory processes. It has been reported that redox reactions are triggered by the presence of free iron, as it can easily accept or donate electrons, favouring the formation of ROS [18]. bLF can scavenge oxygen free radicals and hydroxyl, presenting a potential approach to treat DED [24,25].

However, one of the major challenges of ocular treatment, is the fast elimination via conjunctiva and nasolacrimal duct. It results in a pre-corneal drug half-life of 1–3 min and the need for frequent administrations [26]. In consideration of that, during the last years, drug administration using nanotechnological carriers for controlled release has attracted great interest, owing to improved stability, permeability, and bioavailability, offering advantages over traditional pharmaceutical forms [27,28]. 

Liposomes, since discovery by Bangham [29], have been widely used as delivery system for therapeutic and diagnostic compounds such as drugs, imaging agents, genes, or proteins [30]. Liposomes enhance the active corneal permeability due to their ability to come in close contact with cornea and conjunctiva as well as increase the extent of corneal uptake by prolonging the corneal contact time [31].

Therefore, bLF encapsulation into biocompatible and biodegradable liposomes has been carried out to overcome its compromised stability and increase therapeutic activity and half-life in the ocular surface, granting its prolonged release [31,32]. 

Moreover, one of the most utilised viscosity-building macromolecules in ocular delivery devices is hyaluronic acid (HA), an anionic polysaccharide with ocular mucomimetic properties, that exhibits the capacity of prolonging the precorneal residence time and reducing surface desiccation [3,33].

Therefore, the aim of this study was the development of a nanostructured drug delivery system based on HA-coated bLF-loaded liposomes for the treatment of DED. This study has focused on the incorporation of a high molecular weight protein within a lipidic nanocarrier. Likewise, different in vitro and in vivo studies have been carried out to assess their biocompatibility, capability to reverse DED symptoms, and anti-inflammatory efficacy. Moreover, achievement of sustained drug release and corneal permeability is essential for improving the pharmacokinetic and pharmacodynamic profile of bLF.

## 2. Materials and Methods

### 2.1. Materials

bLF was purchased from Azienda Chimica e Farmaceutica (Fiorenzuola d’Arda, Italy); fat-free soybean phospholipids with 70% phosphatidylcholine (lipoid S75) from Lipoid Gmbh (Ludwigshafen am Rhein, Germany); cholesterol and polysorbate 80 were purchased from Sigma Aldrich (Madrid, Spain); and Sodium hyaluronate was kindly donated by Bloomage Freda Biopharm (Jinan, China). Water filtered through a Millipore^®^ MilliQ system was used for all the experiments and all the other reagents used were of analytical grade.

### 2.2. Lactoferrin Loaded Liposomes Production

bLF loaded liposomes (bLF-LIP) were produced using lipid film hydration method [34]. Briefly, the oil phase was formed dissolving a predetermined amount of lipids (lipoid S75) and cholesterol in 2 mL of ethanol (0.002% tocopherol). Aqueous phase was obtained by dissolving bLF (20 mg × mL^−1^) and polysorbate 80 (P80) (3 mg·mL^−1^) in 10 mL of deionized water. The lipid film was achieved by removing the organic solvent of the oil phase, under reduced pressure, using the rotary evaporation method (Rotavapor^®^ R-210/215 Buchi, Flawil, Switzerland). To ensure complete solvent evaporation, the obtained film was subjected to a nitrogen flow for 10 min. Then, the aqueous phase was added to the lipid film and the mixture was homogenized using an ultrasonic bath (Transsonic Digitals, Elma Schmidbauer GmbH, Singen, Germany). Subsequently, the liposomes undergo a high-pressure homogenization process at 800 mbar at room temperature (2 cycles) by Stansted-pressure cell homogeniser-FPG12800 (Stansted Fluid Power, Harlow, UK). Finally, sodium hyaluronate was added under magnetic stirring to obtain a hyaluronic acid (HA) concentration of 0.1 mg·mL^−1^. 

### 2.3. Optimization of Lactoferrin Loaded Liposomes

A factorial 2^3^ design matrix was employed to obtain the optimal formulation using StatGraphics Centurion XVI.I. This design was established to evaluate the effects of the independents variables (bLF, lipoid S75 and P80 concentrations) on the dependent parameters (average particle size (Z_av_), polydispersity index (PI), zeta potential (ZP) and encapsulation efficiency (EE)) [35]. Each factor was examined at two levels and the responses were modelled through the first-order equation. 

### 2.4. Physicochemical Characterization

Physicochemical parameters such as Z_av_ and PI or ZP were acquired by dynamic light scattering (DLS) and electrophoresis laser doppler, respectively, using a ZetaSizer NanoZS (Malvern Instruments, Malvern, UK). Samples were diluted (1:20) and measurements were carried out by triplicate at 25 °C [36]. 

EE was determined indirectly by quantifying the non-loaded bLF in the dispersion medium. bLF-LIP were ultracentrifugated at 4 °C and 45000 rpm for 60 min and the non-entrapped drug was isolated (Optima^®^ Beckman Coulter, Brea, CA, USA). Then, supernatant was used to evaluate the EE according to the following equation [37]:

(1) $$\mathrm{EE}\left(\%\right)=\frac{\mathrm{Total}\;\mathrm{amount}\;\mathrm{of}\;\mathrm{bLF}-\mathrm{Free}\;\mathrm{amount}\;\mathrm{of}\;\mathrm{bLF}}{\mathrm{Total}\;\mathrm{amount}\;\mathrm{of}\;\mathrm{bLF}}\times 100$$

The amount of the bLF in the aqueous phase was quantified by a reverse-phase high-performance liquid chromatography (RP-HPLC) method described elsewhere [38]. The methodology was validated in accordance with the international guidelines (EMEA, 2011), involving the evaluation of linearity, sensitivity, accuracy, and precision. Concisely, samples were quantified employing HPLC Waters 2695 separation module (Waters, Milford, MA, USA) and a Europa^®^ Protein 300 C_8_ column (5 μm, 250 × 4.6 mm) (Teknokroma Analítica, Barcelona, Spain). Mobile phase was constituted by a water phase containing 0.1% trifluoro acetic acid (TFA) and an organic phase consisting on acetonitrile/water/TFA (95:5:0.1), applying a gradient (from 95% to 25% of water phase and back in 8 min, keeping this ratio up to 25 min) at 0.75 mL·min^−1^. Concentrations ranged from 0.1 to 1 mg·mL^−1^ were used in calibration curve. A diode array detector Waters^®^ 2996 (Waters, Milford, MA, USA) at a wavelength of 219 nm was utilized to identify the bLF and data were handled using Empower 3^®^ Software.

### 2.5. Morphological Characterization and Interaction Studies of Optimized Liposomes

The morphological evaluation of bLF-LIP was done using a Tecnai^®^ G2 F20 TWIN cryogenic transmission electron microscopy (Cryo-TEM) (FEI Company, Hillsboro, OR, USA). Interaction studies were carried out through Differential Scanning Calorimetry (DSC). Thermograms were acquired on a Mettler TA 4000 system (Mettler, Greifensee, Switzerland) equipped with a DSC-25 cell. Samples were weighted in perforated aluminium pans (Mettler M3 Microbalance, Mettler, Greifensee, Switzerland) and heated under nitrogen flow at rate of 10 °C/min. An empty pan with similar attributes was utilized as reference [26]. 

### 2.6. Stability Studies

The stability of bLF-LIP stored at 4 and 25 °C was studied analysing light backscattering (BS) using Turbiscan^®^ Lab (Formulaction, Toulouse, France). Twenty millilitres of sample were introduced into a glass measurement cell. The light source was a pulsed near infrared light source (λ = 880 nm) and it was detected by a BS detector at an angle of 45° from the incident beam. BS data were obtained at 1, 15, 30 and 60 days for 24 h at periods of 1 h. Likewise, measures of Z_av_, PI, ZP, and EE were assayed.

### 2.7. Biopharmaceutical Behaviour 

Direct dialysis bag technique was applied to examine the in vitro release profile due to the hydrophilicity of bLF [39]. bLF-LIP were placed in 1 mL dialysis bags (Float-A-Lyzer^®^ dialysis device, 1000 kDa) (Repligen Corporation, Waltham, MA, USA) and phosphate buffer saline (PBS) 0.1 M buffer solution (pH 7.4) was employed as release medium and maintained under magnetic stirring at 37 °C. At various time intervals, 1 mL of release medium was removed and replaced with fresh buffer solution. RP-HPLC method previously described was used to analyse and data were adjusted to the most frequent pharmacokinetic models [40].

The ex vivo bLF permeation study from bLF-LIP was carried out using isolated corneas from New Zealand rabbits (2.5 kg males), according to the Ethics Committee of Animal Experimentation from the University of Barcelona (CEEA-UB), using a method described elsewhere [26]. Briefly, corneas were placed in a Franz-type cell between donor and receptor compartments. The receptor compartment was filled with PBS at 32 °C, under magnetic stirring. At pre-selected times, 300 µL of sample were withdrawn and replaced by PBS. Samples were directly quantified by RP-HPLC [11,41]. Tests were carried out by triplicate and values were registered as the mean ± SD.

### 2.8. Cytotoxicity 

Human corneal epithelial cells (HCE-2) (LGC Standards, Barcelona, Spain) were used to perform in vitro MTT cytotoxicity assay, previously described [42]. To elucidate the possible cytotoxicity of the formulation, cells were exposed to bLF-LIP and free bLF at different drug concentrations (0.2–2 mg·mL^−1^) for 24 h of incubation. The absorbance was read at λ = 560 nm by an automatic Modulus^®^ Microplate Photometer (Turner BioSystems, Sunnyvale, CA, USA). Viability was expressed as percentage of negative control (untreated cells).

### 2.9. Ocular Tolerance 

To assess ocular tolerance, in vitro HET-CAM test was carried out to guarantee that bLF-LIP was non-irritating after topical administration [43]. Irritation, coagulation, and haemorrhage phenomena in the chorioallantoic membrane of a fertilized chicken egg were evaluated by applying 300 μL of samples. The effects were checked during the first 5 min after the application. Test was performed according to the guidelines of ICCVAM (The Interagency Coordinating Committee on the Validation of Alternative Methods). Eggs (purchased from the farm G.A.L.L.S.A, Tarragona, Spain) were kept at 12 ± 1 °C for at least 24 h before putting them in the incubator with monitored temperature (37.8 °C) and humidity (50–60%) during the incubation days. At day 9 of incubation, 3 eggs were used for each group (free bLF, bLF-LIP, positive control (NaOH 0.1 M) and negative control (0.9% NaCl). Ocular irritation index (OII) was determined by the sum of the scores of each damage parameter according to the expression:

(2) $$\mathrm{OII}=\frac{\left(301-H\right)\times 5}{300}+\frac{\left(301-V\right)\times 7}{300}+\frac{\left(301-C\right)\times 9}{300}+\frac{\left(301-V\right)\times 7}{300}+\frac{\left(301-C\right)\times 9}{300}$$

where H, V, and C are times (s) up to the start of haemorrhage (*H*), vasoconstriction (*V*), and coagulation (*C*), respectively. The formulations were categorized according to the following classification: OII ≤ 0.9 non-irritating; 0.9 < OII ≤ 4.9 weakly irritating; 4.9 < OII ≤ 8.9 moderately irritating; 8.9 < OII ≤ 21 irritating [43,44]. 

To confirm the results acquired from the HEM-CAM test, in vivo Draize test was carried out to evaluate primary ocular irritation [26]. New Zealand male albino rabbits of 2.0–2.5 kg were maintained under monitored ambient conditions with food and water ad libitum. For the experiment, 50 μL of bLF-LIP suspension were applied in the ocular conjunctival sac followed by a slight massage (*n* = 3/group). The appearance of irritation signs was evaluated at the time of instillation and following 1 h. If necessary, evaluation was also carried out at predefined intervals: 24 h, 48 h, 72 h, and 7 days. Draize test score was established by examining the ocular anterior segment and alterations in the structures of the cornea (turbidity or opacity), iris, and conjunctiva (congestion, chemosis, and swelling) (for detailed punctuation see Table A1 of Appendix A). 

### 2.10. Induction and Treatment of Dry Eye 

Induction of moderate dry eye was performed in male New Zealand albino rabbits (2.5 kg). The animals were treated for two weeks with two drops per day of 0.1% benzalkonium chloride in the right eye. Afterwards, the tear level was evaluated throughout Schirmer’s test and the animals were treated for 5 days, either with bLF- LIP or with NaCl 0.9% (positive controls) [45].

Measurement of aqueous tear secretion was carried out using tear strips of Care Group^®^ (Gujrat, India). General anaesthesia was induced to the rabbits using intramuscular ketamine/xylazine (35/5 mg/kg). Subsequently, 0.5% proparacaine (local anaesthetic) was administered topically. The lower eyelid was pulled down slightly and placed the test paper strip on the palpebral conjunctival vesica, which is near the junction of the middle and outer third of the lower lid. The soaked length (in millimeters) of the paper strip was read 5 min later. The procedure was performed by triplicate [45].

### 2.11. Anti-Inflammatory Efficacy Assays

In vitro proinflammatory cytokines determination was assessed to evaluate the anti-inflammatory activity of the bLF-LIP and free bLF in HCE-2 cells. Samples were added to the culture medium at 2 mg·mL^−1^ of bLF and inflammation was induced with LPS (1 μg·mL^−1^). Cells stimulated only with LPS were set as a positive control and untreated cells as a negative control. After 24 h incubation, supernatants were collected and pro-inflammatory cytokine levels (IL-8 and TNF-α) were quantified using the enzyme-linked immunosorbent assay (ELISA) according to manufacturer’s instructions.

In vivo anti-inflammatory effectiveness was carried out throughout the evaluation test for the inflammation prevention ability and the anti-inflammatory efficacy. Assays were carried out using New Zealand male albino rabbits (*n* = 3/group), described previously. The activity of bLF-LIP in comparison with free bLF and NaCl 0.9% (control group) was measured. The inflammation prevention study consisted of the ocular application of 50 μL of each formulation. After 30 min of exposure, an inflammatory stimulus, 50 μL of 0.5% sodium arachidonate (SA) dissolved in PBS, was instilled in the right eye and the left eye was used as a control. In the anti-inflammatory treatment study, the inflammatory stimulus was applied 30 min before than the application of each formulation tested. The evaluation of prevention and treatment of each formulation were carried out from the first application up to 210 min, according to the Draize modified test scoring system (Table A1 of Appendix A) [26].

### 2.12. Statistical Analysis 

Two-way ANOVA followed by Tukey post hoc test was performed for multi-group comparison. Student’s t-test was used for two-group comparisons. All the data are presented as the mean ± S.D. Statistical significance was set at *p* ˂ 0.05 by using GraphPad Prism 8.4.3. ImageJ was used to analyse images.

## 3. Results and Discussion

### 3.1. Optimization Study

**Figure 1:**
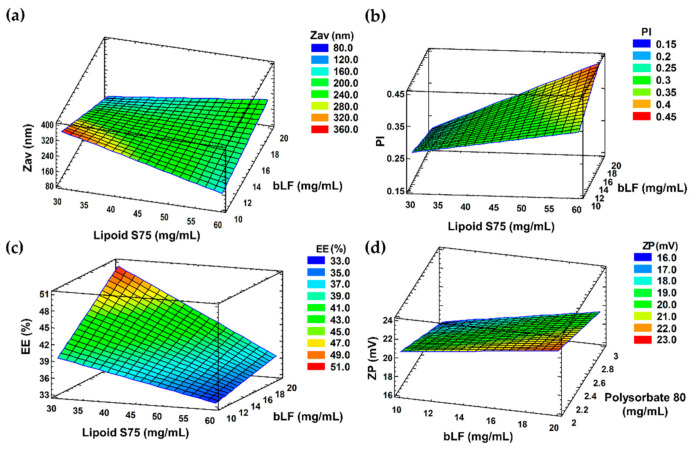
(**a**) Z_av_, (**b**) PI, and (**c**) EE (%) surface response at a fixed P80 concentration (3 mg·mL^−1^). (**d**) ZP surface response at a lipid concentration (30 mg·mL^−1^).

Aiming to achieve the optimal formulation, the effect of independent variables such as concentrations of bLF, lipoid S75 and P80 on the physicochemical properties of the liposomes was evaluated by 2^3^ factorial design. Table 1 shows the results obtained in the optimization study and the corresponding surface responses are showed in Figure 1. 

Concerning Z_av_, PI, and EE, lower lipids concentrations (30 mg·mL^−1^) and higher protein concentrations (20 mg·mL^−1^) favoured smaller particle size, lower PI values, and greater drug encapsulation. Regarding EE and ZP, the most influential variable was lipoid S75 concentration. bLF-LIP obtained had an average size of 85 nm, + 23 mV of ZP, and PI around 0.165, characteristic of a monomodal system, so they are suitable for ocular administration [46]. The PI is a measure of size distribution, agreeing with the literature, liposomal formulation is considered to be heterogeneous if the value is > 0.3 [47]. Cationic liposome formulation improves ocular surface adherence since ocular mucosa depicts slightly negative charge over its isoelectric point, thus increasing ocular bioavailability and prevent tear washout, prolonging corneal residence time [48]. P80 surfactant concentrations showed a slight effect on ZP, being inversely proportional. Increasing the concentration of surfactant resulted in significant particle size reduction. Results are in accordance with those obtained by other authors [49,50]. During the last years, the use of surfactants has been researched for the application in liposomal formulations. P80 is a biodegradable, non-ionic surfactant with great emulsifying properties, generally recommended as safe (GRAS) excipient with established safety profile, without causing ocular irritation [51]. It has reported to be well tolerated in ocular administration up to concentrations of 10% [52]. According to FDA GRAS list, the maximum allowable limit for its use in ophthalmic emulsions is 4% w/w, thus, in the factorial design we have chosen concentration in the range of 0.02–0.03% *w/w* to minimize adverse effects [53]. The addition of P80 decreases the interfacial tension and form smaller emulsion droplets by stabilizing oil/water interface [54].

From the factorial design outcomes, an optimized formulation (F4) was selected. As it can be noted in Table 1, the optimized bLF concentration was 20.00 mg·mL^−1^, 30.00 mg·mL^−1^ of lipoid S75 and 3.00 mg·mL^−1^ of surfactant. The morphometry and surface charge (Z_av_, PI and ZP) were established by photon correlation spectroscopy.

Final optimized formulation, obtained by adding hyaluronic acid (HA) 0.1 mg·mL^−1^ to formulation 4 of factorial design, retained suitable physicochemical properties for ocular administration (Table 2). Z_av_ and PI of the HA coated liposomes increased slightly after HA addition, whereas ZP became less positive. This is due to the fact that HA molecules possess a negatively charged carboxylic acid groups in their chemical structure which are able to interact with cationic liposomes (F4) by electrostatic forces. This caused a decrease in ZP and also led to a tiny increase in Z_av_ and PI [55].

### 3.2. Morphological Characterization 

**Figure 2:**
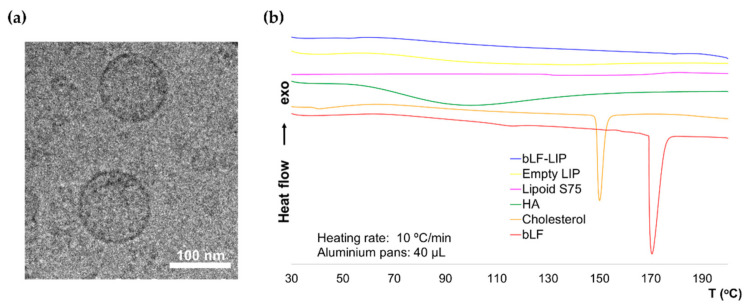
(**a**) Cryo-TEM image of bLF-LIP. (**b**) DSC thermograms of bLF-LIP.

The addition of HA produced a slight increase in average size and PI and a slight ZP reduction, maintaining a strongly positive potential that favours the stability of the system through repulsion by electrostatic forces between particles [56]. Optimized bLF-LIP were characterized morphologically by imaging using cryo-TEM (Figure 2a). Images revealed a spherical and homogenous shape of bLF-LIP, without aggregation events and average particle dispersion similar to the obtained by DLS. According to other authors, the addition of P80 surfactant could contribute to the morphology improvement [54].

### 3.3. Interaction Studies

A factor that considerably influences the pharmacokinetics of the active substance is the physical state of the drug inside the nanosystem. DSC study was carried out to determine the physical state of bLF and the components of the formulation (Figure 2b). The bLF thermogram presents a severe endothermic accident related to its fusion, with a maximum temperature (T_max_) of 170.41 °C which was not found in bLF-LIP. The cholesterol melting peak (T_max_ 150 °C) was also missing. The HA thermogram showed a wide and slight endothermic event around 100 °C, which appeared smoothed in the bLF-LIP thermogram compared with the lipoid S75 one, may be due to the melting of the polymer [57]. Typically, HA presents an exothermic peak at around 240 °C, attributed to the degradation of the polysaccharide. However, it is not observed since it is out of the temperature range, being not relevant to the study [57,58]. Likewise, the lipoid S75 thermogram did not exhibit any thermal events in the range, as a consequence of its low melting point [59]. Empty liposomes showed a similar thermogram to bLF-LIP. Data suggest the adequate incorporation of the formulation components within the bLF-LIP structure. 

### 3.4. Stability of Lactoferrin Loaded Liposomes

**Figure 3:**
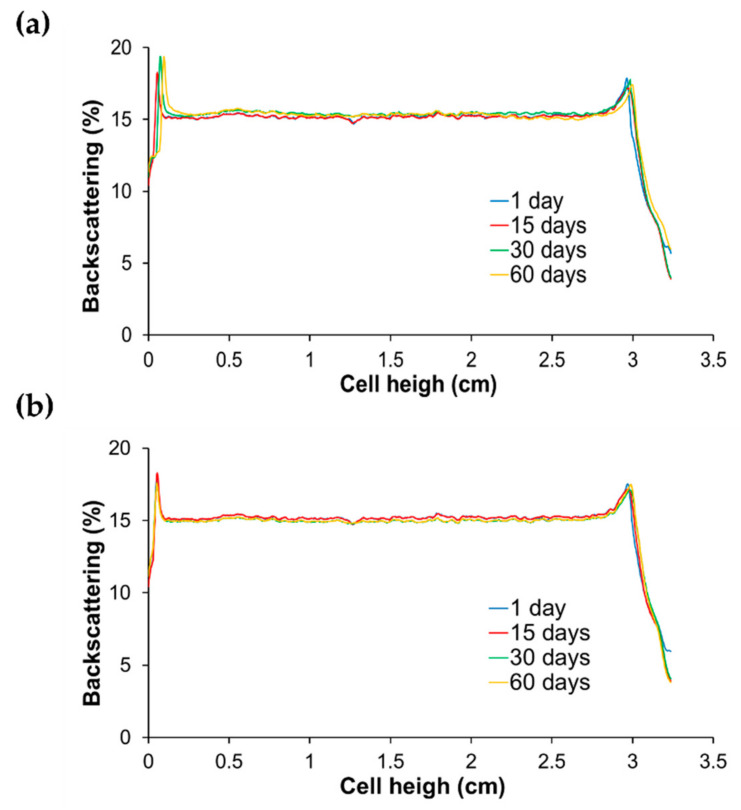
Backscattering profiles of bLF-LIP stored at: (**a**) 4 °C and (**b**) 25 °C.

The BS profile of bLF-LIP was analysed over 60 days (Figure 3). This technique identifies the different destabilization phenomena of the colloidal suspension such as creaming, sedimentation and flocculation or coalescence [60]. The optimized formulation was stored at 4 and 25 °C. BS profile did not show any process of destabilization or migration of particles through the time or fluctuations greater than 5%, which indicates that bLF-LIP remain stable stored at both temperatures. This technique allows predicting the instability courses of liposomes earlier than detected by other methods [28]. Furthermore, several authors have reported that liposomal particle size bellow 90 nm allows for better stability of the colloidal dispersion because gravitational phase partition is avoided by Brownian motion [56]. Moreover, the high value of ZP, over +20 mV, avoids electrostatic interaction between particles and the consequent phenomena of instability [61]. 

### 3.5. Biopharmaceutical Behaviour of bLF-LIP

**Figure 4:**
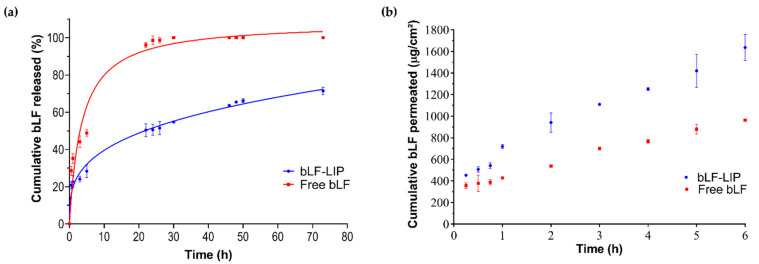
Biopharmaceutical behaviour. (**a**) In vitro release profile of bLF-LIP (Korsmeyer–Peppas equation) against free bLF (hyperbola equation). (**b**) Ex vivo corneal permeation profile of bLF-LIP compared with free bLF and permeation parameters.

The in vitro release of bLF from bLF-LIP and free bLF exhibited a controlled and prolonged release of bLF from nanosystems. The release from liposomes arises as a function of physical and chemical processes that compromise membrane stability carrying out to a few or complete leakage of the liposomal content [62]. Drug release is highly dependent on the composition of the liposomal formulation, including amount of cholesterol, charge, side chains or acyl chain length; and also on the pharmacokinetic properties of the drug itself [62]. Free bLF showed an earlier release, reaching 98% after 24 h, adjusted to hyperbola equation release profile (AIC = 90.60, r^2^ = 0.96), characterized by a rapid release followed by a prolonged release [63]. However, bLF-LIP formulation exhibited a more sustained release. Characterized by a faster bLF release stage, during the first 24 h, up to 50.53%, and afterward, the release speed of bLF-LIP decreased significantly, leading to a prolonged release up to 71.44% after 72 h, without reaching a plateau (Figure 4a). 

The non-linear regression models such as Higuchi or Korsmeyer–Peppas are the two most utilized mathematical models to interpret non-linear diffusion profiles [64]. This biphasic release profile was probably caused by the drug diffusion through the bilayer and HA coating [65]. The Korsmeyer–Peppas release kinetics was the most accurate model to fit the experimental data, showing a minimum Akaike Information Criterion (AIC) value and a maximum r^2^ value (AIC = 67.83, r^2^= 0.98). Data were adjusted to the most common kinetic models to obtain the best fit for bLF release (Table 3).

Ex vivo corneal permeation of bLF-LIP and free bLF were performed to study their behaviour and compare different permeation parameters (Figure 4a). Permeation parameters were obtained by plotting the cumulative bLF permeated versus time, determining the x-intercept by linear regression analysis (Figure 4b) [66]. Table 4 shows that bLF-LIP formulation presents statistically significant differences (*p* < 0.05) against free bLF in all examined permeation parameters. According to the steady-state flux (J) value is twice higher in bLF-LIP, therefore bLF from LIPs infused the cornea faster than free bLF. All permeation parameters follow similar ratios, with the permeability coefficient (K_p_) and the quantity permeated at 24 h (Q24) greater in bLF-LIP than free bLF. Otherwise, the opposite ratio is observed in the case of QR, being twice longer the bLF quantity retained in the cornea from free bLF sample. Owing to their high lipophilicity, the epithelium layer of the cornea, composed of lipid, favoured the release of bLF-LIP and prevents the entry of hydrophilic substances, such as free bLF solution, thus retaining a significant part of the protein in the cornea [67]. Furthermore, particles below 100 nm, particles with deformable nature and positively charged liposomes could potentially facilitate their permeability and absorption through the corneal membrane, leading to an enhance in all pharmacokinetic parameters [31,47]. Therefore, bLF-LIP may efficiently release bLF to the specified area by delivering bLF slowly across the corneal tissue, which would be helpful for the management of DED and the derived ocular inflammation.

### 3.6. Cytotoxicity 

**Figure 5:**
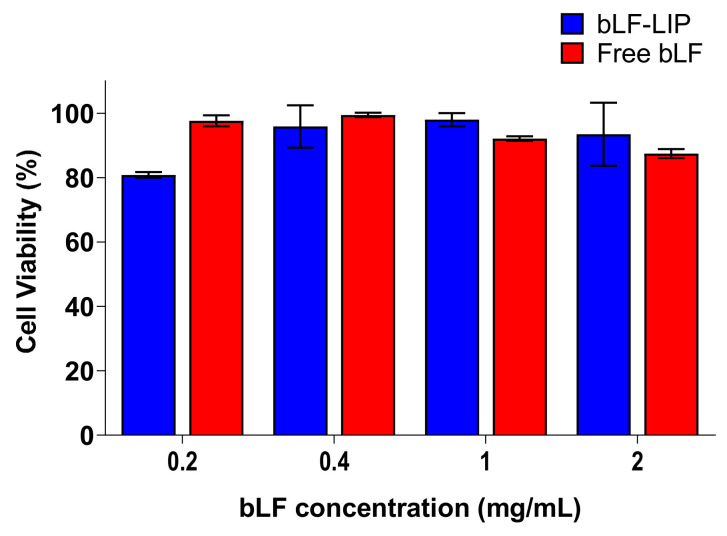
Effect of bLF-LIP on the viability of HCE-2 cells. The 100% cell viability corresponds with the average of MTT reduction values of untreated cells.

To settle liposomes ocular administration suitability, in vitro MTT cytotoxicity assay was evaluated in HCE-2 cells (Figure 5). Results showed that after 24 h incubation both bLF-LIP and free bLF did not cause relevant cytotoxic effects. Cell viability was higher than 80% at all concentrations tested. 

LF is one of the most abundant components in the healthy tear fluid, representing 20–30% of the total proteins, varying between 0.63–2.9 mg·mL^−1^, depending on gender and age [68,69]. The basal tear flow (1 μL·min^−1^) is considerably increased upon acute stimulation, the expression of LF is upregulated to inhibit the production of inflammatory cytokines [18]. Moreover, LF contributes to antimicrobial activity via inhibiting the growth of bacteria and mitigates oxidative stress via iron retention mechanism [5,16]. However, current research has proved that these activities are inactive in DED patients [70]. It is due to the fact that there is a reduced LF amount at ocular level, since tear volumes have positive correlation with LF concentration [18]. Hence bLF-LIP could provide a sustained release of protein and improve its bioavailability, for cases in which LF tear concentration is compromised.

Regarding other formulation components, it has been reported that lipoid s75 (soybean phospholipids with 70% phosphatidylcholine) is non-immunogenic, biocompatible, biodegradable and a safe substance used for the development of lipid vehicles for delivering pharmacological substances with a broad range of solubilities at ocular level [62,71].

HA is widely used in the management of DED. Is a naturally occurring, endogenous, glycosaminoglycan polymer present in various tissue fluids in human body, mainly in the extracellular matrix [72]. In particular, high molecular weight HA (>1000 kDa) was reported to have some immunosuppressive, antioxidant, anti-inflammatory, anti-angiogenic effect, and wound repair capacity [73]. 

These outcomes verify the biocompatibility of the developed bLF-LIP with corneal cells, matching with the generally recognized as safe (GRAS) designation of the formulation components [71]. 

### 3.7. Ocular Tolerance

**Figure 6:**
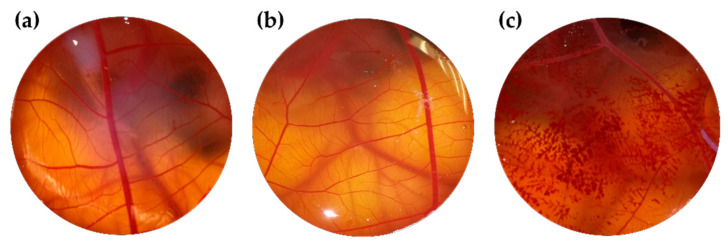
HET-CAM test assessed with different formulations: (**a**) bLF-LIP; (**b**) free bLF and (**c**) positive control.

In vitro ocular tolerance was studied by HET-CAM test. bLF-LIP and free bLF were proved to verify the potential instant irritation response in the CAM of 3 eggs. The addition of free bLF solution or bLF-LIP did not reveal any sign of damage or vascular alteration. Likewise, negative control (0.9% NaCl) did not produce any response over the time tested. In contrast, the addition of positive control (1M NaOH) generated a severe vasoconstriction and haemorrhage [74]. As shown in Figure 6, the suitability for ocular administration is confirmed. The outcome showed that, at the ocular level, bLF-LIP are classified as a non-irritating substance (Table 5). These results agree to those obtained by in vitro HCE-2 cytotoxicity assays.

In vivo ocular tolerance Draize test or primary irritation test was assessed to verify the irritation potential of the optimized liposomes formulation [75]. Rabbit model is commonly chosen to perform these experiments because its ocular physiology is well known and implies easy manipulation. Moreover, rabbit eye is usually more susceptible to irritation than the human eye [76]. Due to the high sensibility of the ocular surface, it was essential to check possible irritating effects or ocular damage caused [26].

The in vivo test was assessed considering taking into account that each of the formulation compounds was safe and biocompatible, based on the previously performed in vitro HET-CAM test and analysed by other authors [48,77]. 

Results showed no signs of redness, ocular inflammation, or increased tear production following instillation of bLF-LIP, being the total score for each rabbit zero (Table 5). Therefore, bLF-LIP could be classified as non-irritant substance. 

### 3.8. Therapeutic Efficacy against Dry Eye Disease

**Figure 7:**
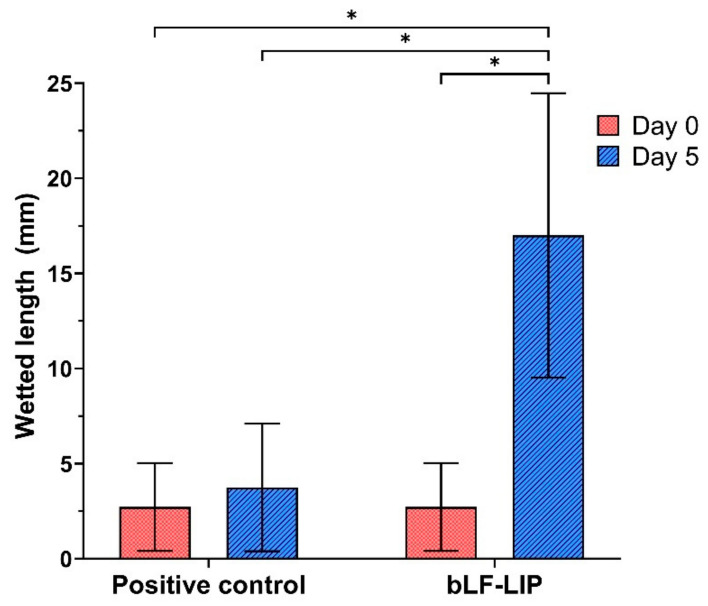
Schirmer’s test results. Values are expressed as mean ± SD; * *p* < 0.05 significantly higher than the secreted tear by non-treated with bLF-LIP eye for 5 days.

Aiming to verify the therapeutic efficacy of the developed bLF-LIP in the treatment of dry eye, the Schirmer’s test was carried out. A severe decrease in the aqueous tear secretion was achieved after the application of benzalkonium chloride for 2 weeks. Figure 7 showed a considerable difference between the tear volume secreted by the dry eye of positive control and by the group treated with liposomes. There were statistically significant differences on days 0 and 5 in bLF-LIP group, being 6.25-fold higher after 5 days of treatment, and 4.5-fold greater than the eye treated with physiological saline. These results matched with the result obtained by other authors. One of these studies reported an ameliorated dry eye symptoms and tear film stability in patients supplemented with oral LF [78]. Furthermore, these results are supported by other authors that studied the ocular instillation of LF in a rabbit dry eye model, resulting in a restoration of corneal epithelial integrity, suggesting its potential use for treating DED [77]. In the case of the developed bLF-LIP formulation, improved drug pharmacokinetics and pharmacodynamics were observed thanks to its encapsulation within biocompatible lipidic nanosystem.

### 3.9. Anti-Inflammatory Efficacy 

#### 3.9.1. In Vitro Assays: IL-8 and TNF-α Determination

**Figure 8:**
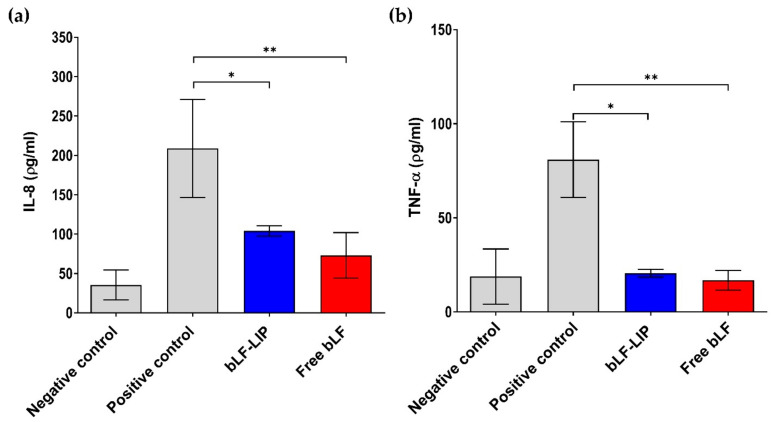
(**a**) Quantification of secreted IL-8 proinflammatory cytokine in LPS-stimulated HCE-2 cells; (**b**) quantification of secreted TNF-α. Negative control: no treatment; Positive control: LPS. Values are expressed as the mean ± SD; * *p* < 0.05; ** *p* < 0.01 significantly lower than LPS-induced cytokine concentration.

There is evidence that in the dry eye syndrome and chronic inflammation-associated, tears present overexpression of different inflammatory mediators, specially IL-8 and TNF-α cytokines [79]. Hence, the in vitro cytokines determination was carried out in HCE-2 cells to assess the ability of bLF-LIP to inhibit the inflammatory response caused by LPS (Figure 8a,b) [80]. 

Various authors have studied the influence of DED on the presence of different inflammation markers at ocular level. It has been reported that there is a significant increase of inflammation, doubling the concentration of IL-8 in patients with DED compared with healthy controls [2,81]. This high concentration of IL-8 at the tear level leads to the migration of different immune cells towards the eye, triggering the aggravation of the ocular inflammation symptoms present in the disease [15]. At the same time, higher tear concentration of another inflammatory cytokine, TNF-α, had been detected, keeping the inflammatory environment in patients with different ophthalmopathies-associated [79,81,82].

In Figure 8 it can be observed that the highest levels of cytokines induced by LPS were obtained in the absence of bLF-LIP (positive control). Administration of bLF-LIP considerably diminished the expression of IL-8 and TNF-α, reaching similar levels to those obtained with free bLF (*p* < 0.05). This fact indicated that an anti-inflammatory effect was achieved with the administration of bLF-LIP in corneal cells.

The findings are in accordance with what has been described for LF, which has the capacity to modulate the expression of various cytokines through different mechanisms [77]. Including the interaction with cell surface receptors involved in the inflammatory response, by binding to CD14 receptor, thus diminishing NF-κB-induced transcription of various genes encoding inflammatory mediators [13,21,83]. Regarding to its iron-chelating ability, LF can manage the oxidative burst produced by neutrophils and macrophages, by oxygen free radical and hydroxyl scavenging activities, thus mitigating the inflammatory response and tissue damage caused by ROS [19].

#### 3.9.2. In Vivo Assays

In vivo anti-inflammatory efficacy was assayed to confirm the capacity of the liposomes to prevent and treat ocular inflammation through two different tests. 

**Figure 9:**
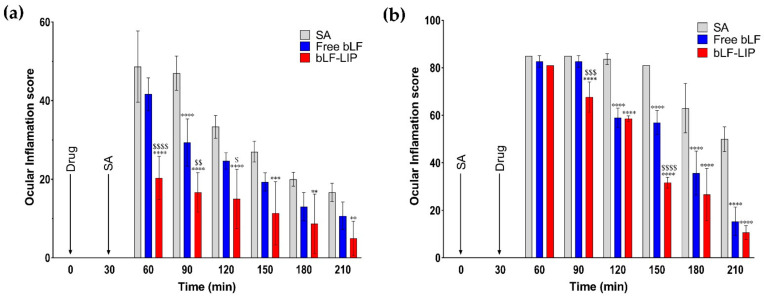
(**a**) Ocular inflammation prevention. (**b**) Ocular inflammation treatment test. Values are expressed as mean ± SD; * *p* < 0.05, ** *p* < 0.01 and *** *p* < 0.001 and **** *p* < 0.0001 significantly lower than the inflammatory effect induced by SA; $ *p* < 0.05, $$ *p* < 0.01 and $$$ *p* < 0.001 and $$$$ *p* <0.0001 significantly lower than the inflammatory effect induced by free bLF.

In vivo inflammatory prevention test showed significant differences between the degree of inflammation treated with bLF formulations or physiological serum during all the timepoints tested. Nevertheless, eyes treated with bLF-LIP presented a faster swelling reduction rather than free bLF, mainly owing to tear clearance in case of free bLF and the improved ocular surface adherence of liposomes, thus presenting longer residence time in the cornea [26]. bLF-LIP exhibited significant differences regarding positive control over the time. Thus, bLF-LIP exhibited a preventive effect of inflammation caused by the sustained release of bLF to the corneal cells (Figure 9a).

In addition, the in vivo inflammation treatment was assessed. Liposomes and free bLF were applied after 30 min of SA exposure, and the degree of inflammation was quantified. 

Figure 9b revealed that the degree of inflammation was significantly reduced after the first hour post-administration of bLF-LIP. This fact confirms its controlled bLF release from liposomes, providing a longer anti-inflammatory activity and enhancing its bioavailability. The presence of a cationic surface charge in the lipidic nanocarrier, may increase the residence time by interaction with the negatively charged corneal epithelium and the mucins from tears fluid and conjunctiva [56]. Moreover, statistically significant differences were observed between the positive inflammation control and the group treated with free bLF, displaying its anti-inflammatory activity [19]. After 90 min of contact, both bLF formulations were effective in treating inflammation symptoms. However, a greater and faster reduction was observed in the case of bLF-LIP during the assay. Hence, it can be concluded that the controlled release system based on bLF-LIP has ocular anti-inflammatory activity, both for prevention level and inflammation treatment.

## 4. Conclusions

In summary, a novel nanotechnological tool has been developed for the management of DED and its ocular complications. It is based on the encapsulation of bLF, an anti-inflammatory and antioxidant high molecular weight protein, into hyaluronic acid coated liposomes. This nanosystem has been proven to be physically stable with a prolonged bLF release as well as high corneal permeability, thus improving biopharmaceutical bLF behaviour. In addition, in vitro and in vivo tests corroborate that the developed formulation is biocompatible without any sign of ocular irritation or cytotoxicity. Furthermore, bLF-LIP exert the ability to revert DED symptoms by restoring physiological tear levels. At the same time, bLF-LIP were able to decrease inflammation both in vitro and in vivo. Hence, hyaluronic acid coated bLF-loaded liposomes constitute a suitable system to treat and prevent DED and ocular inflammation. 

## 5. Patents

Liposomes described in this work have been patented under the reference EP 3603621 A1 and this patent has recently been extended to the US under the reference US 10,835,494 B2.

## Figures and Tables

**Table 1:** Values of the 2^3+^ star central composite rotatable factorial design, parameters and measured responses. Results presented as mean ± standard deviation.

	Independent Variables						Dependent Variables			
	cP80		cbLF		cLipoid-S75		Z_av_	PI	ZP	EE
	(mg·mL^−1^)		(mg·mL^−1^)		(mg·mL^−1^)		(nm)		(mV)	(%)
1	−1	2.0	−1	10.0	−1	30.0	253.6 ± 2.2	0.121 ± 0.024	21.9 ± 0.6	49.2 ± 0.9
2	1	3.0	−1	10.0	−1	30.0	378.2 ± 1.4	0.317 ± 0.064	16.3 ± 0.3	40.6 ± 0.3
3	−1	2.0	1	20.0	−1	30.0	160.0 ± 3.9	0.179 ± 0.021	22.9 ± 0.2	55.4 ± 1.7
4	1	3.0	1	20.0	−1	30.0	85.0 ± 2.4	0.165 ± 0.033	22.7 ± 0.3	50.0 ± 2.5
5	−1	2.0	−1	10.0	1	60.0	471.7 ± 2.3	0.383 ± 0.046	24.3 ± 1.9	39.6 ± 4.0
6	1	3.0	−1	10.0	1	60.0	133.7 ± 1.4	0.292 ± 0.036	25.4 ± 0.6	33.1 ± 1.5
7	−1	2.0	1	20.0	1	60.0	602.8 ± 6.2	0.282 ± 0.016	26.0 ± 1.2	35.3 ± 0.4
8	1	3.0	1	20.0	1	60.0	242.1 ± 2.3	0.484 ± 0.031	26.2 ± 0.3	37.4 ± 0.3

**Table 2:** Physicochemical parameters of bLF-LIP after adding HA.

Z_av_	PI	ZP	EE
(nm)		(mV)	(%)
90.5 ± 0.6	0.201 ± 0.070	20.5 ± 0.4	50.0 ± 3.0

Values are expressed as mean ± standard deviation.

**Table 3:** Parameters for kinetic models of bLF-NPs and free bLF solution.

Models	bLF-NPs		Free bLF	
	AIC	R^2^	AIC	R^2^
Zero Order	94.76	0.84	115.87	0.64
First Order	94.33	0.84	93.67	0.94
Higuchi	77.93	0.96	104.86	0.86
Hyperbola	89.26	0.90	90.60	0.96
Korsmeyer–Peppas	*n* = 0.014		*n* = 0.022	
	67.83	0.98	93.91	0.94

**Table 4:** Pharmacokinetic parameters adjusted to linear regression of the ex vivo corneal permeation of bLF-LIP against bLF.

Parameters	Free bLF	bLF-LIP
J (µg·h^−1^·cm^−2^)	171.79 ± 9.83	317.50 ± 67.84 *
Kp · 10^3^ (cm·h^−1^)	8.59 ± 0.49	15.88 ± 3.39 *
Q24 (µg)	2635.83 ± 151.49	4874.52 ± 1042.71 *
QR (µg·g^−1^·cm^−2^)	1.12 ± 0.01	0.55 ± 0.02 **

Statistical significance: * *p* < 0.05, ** *p* < 0.0001. J, steady-state flux; Kp, permeability coefficient; Q24, permeated amount at 24 h; QR, retained amount.

**Table 5:** Ocular tolerance: in vitro (HET-CAM) and in vivo (Draize test).

Formulation	Medium Score		Classification
	HET-CAM	Draize	
bLF-LIP	0.07 ± 0.00	0.00 ± 0.00	Non-irritant
Free bLF (20 mg·mL^−1^)	0.07 ± 0.00	0.00 ± 0.00	Non-irritant

## Appendix A

**Table A1:** Rating scale to evaluate the degree of ocular irritation (Draize test).

Structure	Injury	Evaluation	Score
CORNEA	(A) Degree of cloudiness or opacity		Corneal score: A × B × 5 Maximum score: 80
	-Absence of ulceration	0	
	-Diffuse areas	1	
	-Translucent areas	2	
	-Opalescent areas	3	
	-Full opacity	4	
	(B) Affected areas		
	-None	0	
	-A quarter or less	1	
	-More than a quarter but without means	2	
	-More than three quarters up a whole plane	3	
	-More than half but less than three quarters	4	
IRIS	(A) Iris injury score		Iris score: A·× 5 Maximum score: 10
	-Normal	0	
	-Deep folds, congestion, swelling, moderate circumcorneal injection	1	
	-No reaction to light, haemorrhage, great destruction	2	
CONJUNCTIVA	(A) Redness		Conjunctival score: (A + B + C) × 2 Maximum score: 20
	-Normal glasses	0	
	-Some clearly injected vessels	1	
	-Diffuse redness	2	
	-Big diffuse redness	3	
	(B) Chemosis or Inflammation		
	-None	0	
	-Some	1	
	-Marked with partial disorder of the eyelids	2	
	-Eyelid more or less closed	3	
	-Semi eyelids	4	
	(C) Sweat		
	-None	0	
	-Any amount anomalous	1	
	-Wetting and eyelid hairs	2	
	-Periocular wetting	3	
Calculation of the Ocular Irritation Index		OII	Classification
OII = Cornea (A·× B·× 5) + Iris (A × 5) + Conjunctiva ((A + B + C) × 2)		0	Non-irritant
		0–15	Weakly irritant
		>15–30	Moderately irritant
		>30–50	Irritant
		>50	Extremely irritant
